# Association of urinary non-albumin protein with the different urinary marker for glomerular and tubular damage in patients with type 2 diabetes

**DOI:** 10.1186/s12882-020-01906-6

**Published:** 2020-07-06

**Authors:** Khalid Siddiqui, Salini Scaria Joy, Shaik Sarfaraz Nawaz, Dhekra Alnaqeb, Muhammad Mujammami, Khalid Al-Rubeaan

**Affiliations:** 1 Strategic Center for Diabetes Research, College of Medicine, King Saud University, Riyadh, Saudi Arabia; 2 University Diabetes Center, King Abdulaziz University Hospital, King Saud University, Riyadh, Saudi Arabia; 3 Endocrinology and Diabetes, Department of Medicine, King Khalid University Hospital & College of Medicine, King Saudi University, Riyadh, Saudi Arabia

**Keywords:** Non-albumin protein, Total protein, Transferrin, Retinol-binding protein, Neutrophil gelatinase-associated lipocalin, Urinary markers, Tubular markers and glomerular marker

## Abstract

**Background/aim:**

In recent years, the diagnostic utility of urinary protein levels has been demonstrated for the early detection and progression of kidney disease. This study aimed to evaluate the associations of the non-albumin protein (NAP) with different urinary marker for tubular and glomerular damage in patients with type 2 diabetes (T2D).

**Methods:**

In this observational cross-sectional study, 424 patients with T2D duration > 10 years were classified into two groups according to estimated glomerular filtration rate (eGFR). The ratios of different urinary markers (albumin, NAP, total protein, transferrin, retinol-binding protein (RBP), and neutrophil gelatinase-associated lipocalin (NGAL) to creatinine were analyzed.

**Results:**

The levels of urinary biomarkers increased significantly with decrease in eGFR levels. In the group with moderately decreased eGFR, the albumin to-creatinine ratio (ACR), non-albumin protein-to-creatinine ratio (NAPCR), and total protein-to-creatinine ratio (PCR) were independently associated with all urinary markers after being adjusted for risk factors. The area under the receiver operating characteristics (ROC) curve for ACR and PCR had a better diagnostic value than other urinary biomarkers. Comparing ROC curve of NAPCR with other urinary biomarkers, it was significantly better than NGAL/Cr (*p* = 0.033).

**Conclusions:**

The findings of the present study confirm that ACR and PCR are diagnostic biomarkers in T2D patients with decreased eGFR. NAPCR in these patients diagnostically only outperformed NGAL/Cr.

## Background

Diabetes mellitus (DM) is a growing public health problem throughout the world. In 2017, 425 million people worldwide were estimated to have diabetes, and the number of cases is predicted to increase to 629 million by 2045. Diabetic nephropathy (DN) is one of the major microvascular complications of diabetes and represents the leading cause of end-stage kidney disease. The prevalence of diabetes among the Saudi population has been reported to be 17.7%, and 40% of end stage renal diseases in hemodialysis patients is caused by diabetic nephropathy [1, 2].

Proteinuria is a condition characterized by the presence of increased amounts of protein in the urine, and it is an important indicator of kidney disease in diabetic patients. The condition can be classified on the basis of the type of protein that is detected (albuminuria or non-albuminuria) or the underlying pathological damage (glomerular, tubular or overflow) [3]. Urinary protein comprises both non-albumin proteins (NAPs) and albumin. NAPs are low-molecular-weight proteins including mucoproteins (mainly Tamm-Horsfall protein), blood-group proteins, immunoglobulins, mucopolysaccharides, hormones, and enzymes [4]. Urinary albumin is the most abundant protein found in urine [5]. Increased albumin in the urine is indicative of glomerular proteinuria whereas tubular and overflow proteinuria cannot be characterized by albuminuria alone [6]. Similarly, transferrin is also considered as glomerular marker that is found to significantly increase with progression of diffuse glomerular lesions and is expected to be excreted before the development of microalbuminuria [7]. Recently, many urinary markers including retinol binding protein (RBP) and neutrophil gelatinase-associated lipocalin (NGAL) have been evaluated to predict tubular damage in diabetic patients [8].

In current practice, the albumin-to-creatinine ratio (ACR) is the most commonly used and well-standardized biomarker for the diagnosis of kidney disease in diabetic patients, and several studies have reported the clinical significance of urinary total protein, transferrin, RBP and NGAL levels in DN [8–10]. Methvan et al. reported that the total protein-to-creatinine ratio (PCR) is a more sensitive biomarker for the prediction of proteinuria in chronic kidney disease [11]. The diagnostic utility of urinary total protein in chronic kidney disease as well as in DN has been proven [9, 12, 13]. There are few studies reporting the clinical significance of urinary NAP levels for decreased eGFR levels in type 2 diabetes (T2D). The elevated NAP in the urine has been demonstrated to be an indication of tubular proteinuria and has been significantly associated with various biomarkers of tubular damage [3, 14]. Most of the previous studies did not consider total protein levels in their analyses yet concluded that NAP is a better predictor of renal impairment than other urinary biomarkers [14, 15]. Furthermore, no previous studies have analyzed the correlations between total protein with NAP levels and different urinary biomarkers in T2D with decreased eGFR levels. Therefore, this study, aimed to evaluate the association between the NAPCR with PCR and markers of tubular and glomerular damage, and to compare the diagnostic value of different urinary biomarkers in T2D with reduced eGFR levels.

## Methods

### Study population

This is a cross-sectional study conducted at University Diabetes Center, King Saud University between 1 April 2014 and 18 June 2015. The study was approved by the Institutional Review Board (IRB) of the King Saud University and was conducted in accordance with the Declaration of Helsinki [16]. Written informed consent was obtained from each patient involved.

T2D patients aged between 35 and 70 years and with diabetes duration > 10 years were recruited for this study. Patients with diabetic complications including vasculopathy and retinopathy, or other associated diseases such as hypertension and hyperlipidemia, were included in this study, as these complications are more prevalent among T2D patients having longer duration of diabetes. Diabetes was managed with oral antidiabetic therapy with or without insulin therapy. Hypertension was managed with medications including angiotensin II receptor antagonists, thiazide diuretics, angiotensin-converting enzyme inhibitors, beta blockers and calcium channel blockers. Hyperlipidemia was managed with statins. Details of the exclusion and inclusion criteria have been reported in a previous study [17].

**Fig. 1:**
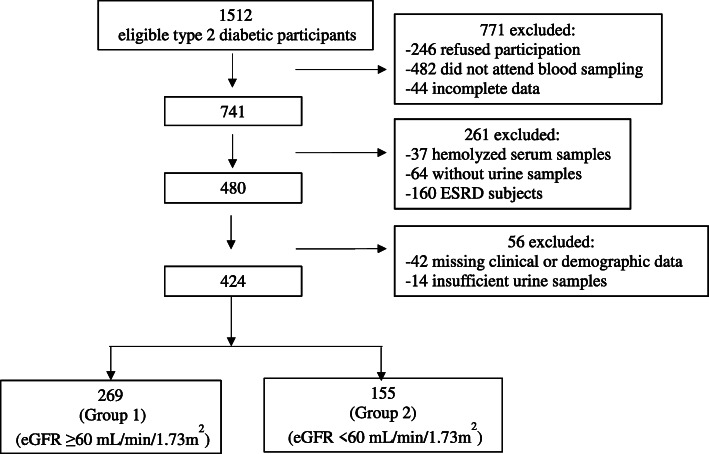
Flow diagram of participants through the study according to STARD criteria

In accordance with the Standards for Reporting Diagnostic Accuracy Studies (STARD), a diagram illustrating the flow of participants through the study is shown in Fig. 1 [18]. Kidney disease in T2D was defined based on the eGFR value <60 mL/min/1.73 m^2^ [19]. A total of 424 T2D patients were selected for this study. The selected patients were divided into two different groups by eGFR: Group 1 (eGFR: ≥60 mL/min/1.73 m^2^), Group 2 (eGFR: <60 mL/min/1.73 m^2^).

### Data collection

After overnight fasting, 5 mL of venous blood was collected from each patient in a plain tube. Serum was separated and immediately stored at −20 °C for further analysis. Biochemical assessment of fasting blood sugar (FBS), HbA1c, serum creatinine, and lipid levels (triglyceride, high-density lipoprotein (HDL), low-density lipoprotein (LDL) and total cholesterol (TC)) were carried out using the Randox RX Daytona Clinical Chemistry Analyzer (Randox, UK).

Body mass index (BMI) was calculated by dividing weight (kg) by height squared (m^2^). Systolic blood pressure (SBP) and diastolic blood pressure (DBP) were measured using a digital blood pressure monitor. Random spot urine samples were obtained from patients during their clinic visits. Urine samples were stored at −80 °C. Urinary albumin, creatinine, and total protein levels were analyzed using the Randox RX Daytona Clinical Chemistry Analyzer. Urinary albumin excretion was estimated by calculating the ACR in units of mg/g, and urinary total protein excretion was estimated by calculating the PCR in units of mg/g. The NAPCR (mg/g) was calculated by subtracting the ACR value from the PCR [16]. The eGFR was calculated using the CKD-EPI creatinine equation (2009) [20].

### Biomarker assays

The levels of urinary biomarkers (transferrin, RBP and NGAL) were measured by solid phase enzyme-linked immunosorbent assay (ELISA) using commercially available standard kits designed for urine analysis (Abcam, Cambridge, MA, USA).

### Statistical analysis

All statistical analyses were performed using SPSS version 21.0 (SPSS Inc, Chicago, IL, USA). Data are presented as mean ± standard deviation for normally distributed variables and as median (interquartile range (IQR)) for skewed variables. Pearson or Spearman correlation analyses were performed using either ACR, PCR, or NAPCR as the dependent variable. For multiple linear regression analysis, either ACR, PCR, or NAPCR was considered as the dependent variable and other urinary markers were considered independent variables. Several models were built to adjust for confounding factors of kidney disease including age, gender, duration of diabetes, systolic blood pressure (SBP), HbA1c, LDL, and eGFR. Receiver operating characteristics (ROC) curves were plotted, and the area under the curve (AUC) was evaluated for different markers. The comparison of ROC curves of different urinary markers using online calculator https://www.medcalc.org/manual/comparison_of_roc_curves.php A *p* value <0.05 was considered statistically significant.

## Results

Table 1 summarizes the clinical and biochemical characteristics of patients with T2D according to eGFR. The number of patients categorized into each of the two groups was as follows: Group 1, 269 (45.0 % males); Group 2, 155 (43.9% males). The age and duration of diabetes significantly increased with decreasing eGFR. There were no significant differences between the two groups with regards to gender, BMI, DBP or LDL. The HbA1c and FBS levels were significantly different between the groups, with HbA1c levels being higher in Group 2 (10.76 %) than Group 1 (10.31 %) (*p* = 0.012). The PCR, ACR, NAPCR, transferrin -to- creatinine ratio (Transferrin/Cr), retinol binding protein-to-creatinine ratio (RBP/Cr) and neutrophil gelatinase-associated lipocalin-to-creatinine ratio (NGAL/Cr) values increased as eGFR decreased and showed a linear trend (*p* < 0.001). The median NAPCR of Group 1 was 88.15 mg/g, compared with 186.13 mg/g for Group 2 (*p* < 0.001).

**Table 1:** Clinical and biochemical characteristics of type 2 diabetes subjects categorized according to eGFR

Variables	Group 1 eGFR (≥60 mL/min/1.73 m^**2**^)	Group 2 eGFR (< 60 mL/min/1.73 m^**2**^)	***p*** value
N	269	155	
Age, years	54.67 ± 6.04	56.08 ± 6.54	0.026
Male gender (%)	45.0	43.9	0.840
BMI, kg/m^2^	32.27 ± 5.53	32.49 ± 6.00	0.703
Duration of diabetes, years	17.84 ± 5.26	19.10 ± 6.16	0.026
SBP, mmHg	134.54 ± 18.26	141.50 ± 21.00	<0.001
DBP, mmHg	73.79 ± 10.50	73.89 ± 11.50	0.927
FBS, mg/dL	207.04 ± 87.01	234.44 ± 101.64	0.004
HbA1c, %	10.31 ± 1.59	10.76 ± 2.08	0.012
Total cholesterol, mg/dL	175.81 ± 39.05	198.83 ± 55.03	<0.001
LDL, mg/dL	132.06 ± 40.11	138.29 ± 51.43	0.169
HDL, mg/dL	45.31 ± 11.07	47.81 ± 13.60	0.041
Triglycerides, mg/dL	166.46 ± 80.12	203.57 ± 87.33	<0.001
ACR, mg/g median (IQR)	15.59(5.96–57.67)	167.50(50.66–634.85)	<0.001
PCR, mg/g median (IQR)	118.70(68.32–238.59)	297.37(155.54–1122.93)	< 0.001
NAPCR, mg/g median (IQR)	88.15(50.89–181.95)	186.13(75.09–613.93)	<0.001
Transferrin/Cr, μg/g median (IQR)	348.87(140.82–1021.62)	1334.19(422.58–3117.02)	<0.001
RBP/ Cr, μg/g median (IQR)	315.67(202.93–503.68)	572.96(305.82–1589.01)	<0.001
NGAL/Cr, μg/g median (IQR)	23.47(11.64–48.42)	36.48(15.83–89.87)	<0.001

Group 1,eGFR ≥60 mL/min/1.73 m^2^; Group 2, eGFR <60 mL/min/1.73 m^2^; *BMI* Body mass index, *SBP* Systolic blood pressure, *DBP* Diastolic blood pressure, *FBS* Fasting blood sugar, *HbA1c* Hemoglobin A1c, *LDL* Low-density lipoprotein, *HDL* High-density lipoprotein, *ACR* Albumin-to-creatinine ratio, *PCR* Total protein-to-creatinine ratio, *NAPCR* Non-albumin protein -to-creatinine ratio, *Transferrin/Cr* Transferrin-to-creatinine ratio, *RBP/Cr* Retinol binding protein-to-creatinine ratio, *NGAL/Cr* Neutrophil gelatinase-associated lipocalin-to-creatinine ratio, *eGFR* Estimated glomerular filtration rate. Data are presented as mean ± standard deviation for parametric variables and median (IQR; Inter quartile range) (25th and 75th) for non-parametric variables and compared by ANOVA. Categorical data are presented as absolute frequencies and compared using the Chi-square test. Values of *p* < 0.05 were considered significant

The relationships between NAPCR and urinary markers as well as clinical and biochemical parameters of each group are summarized in Table 2. The NAPCR was significantly correlated with urinary markers in all patients as well as among both groups. Additionally, the NAPCR was found to be correlated with SBP, DBP and HbA1c level in the patients having eGFR ≥60 mL/min/1.73 m^2^.

**Table 2:** Correlation of non-albumin protein to creatinine ratio with clinical, anthropometric and biochemical characteristics; and other urinary markers in type 2 diabetes subjects

Variables	Total patients (424)		Group 1 (269)		Group 2 (155)	
	r	*p*	r	*p*	r	*p*
Age years	0.082	0.113	0.044	0.480	0.049	0.598
BMI, kg/m^2^	0.042	0.418	0.101	0.111	−0.021	0.826
Duration of diabetes, years	0.138	0.008	0.158	0.011	0.101	0.276
SBP, mmHg	0.136	0.008	0.224	<0.001	0.028	0.766
DBP, mmHg	−0.007	0.889	0.193	0.002	−0.179	0.052
FBS, mg/dL	0.058	0.263	0.056	0.377	0.002	0.980
HbA1c, %	0.191	<0.001	0.212	0.001	0.136	0.141
Total cholesterol, mg/dL	0.079	0.128	0.084	0.180	−0.033	0.727
LDL, mg/dL	−0.002	0.966	0.047	0.456	−0.074	0.432
HDL, mg/dL	0.044	0.394	0.099	0.116	−0.026	0.778
Triglycerides, mg/dL	0.061	0.244	0.022	0.726	0.012	0.898
eGFR, mL/min/1.73 m^2^	−0.411	< 0.001	− 0.151	0.015	− 0.545	< 0.001
ACR, mg/g	0.624	<0.001	0.411	<0.001	0.635	<0.001
PCR, mg/g	0.873	<0.001	0.850	<0.001	0.867	<0.001
Transferrin/Cr, μg/g	0.439	<0.001	0.276	<0.001	0.447	<0.001
RBP/Cr, μg/g	0.555	<0.001	0.516	<0.001	0.539	<0.001
NGAL/Cr, μg/g	0.390	<0.001	0.241	<0.001	0.466	<0.001

Group 1,eGFR ≥60 mL/min/1.73 m^2^; Group 2, eGFR <60 mL/min/1.73 m^2^; *BMI* Body mass index, *SBP* Systolic blood pressure, *DBP* Diastolic blood pressure, *FBS* Fasting blood sugar, *HbA1c* Hemoglobin A1c, *LDL* Low-density lipoprotein, *HDL* High-density lipoprotein, *eGFR* Estimated glomerular filtration rate, *ACR* Albumin-to-creatinine ratio, *PCR* Total protein-to-creatinine ratio, Transferrin/Cr transferrin-to-creatinine ratio; *RBP/Cr* Retinol binding protein-to-creatinine ratio, *NGAL/Cr* Neutrophil gelatinase-associated lipocalin-to-creatinine ratio, r; coefficients of correlation, *p* < 0.05 was considered significant

The relationships of the PCR and ACR with urinary markers as well as clinical and biochemical parameters are summarized in Additional file 1, Table S1 and Additional file 2, Table S2 respectively. Correlations of the PCR and ACR value with urinary biomarkers were similar to those observed for NAPCR in both groups except for NGAL for ACR in Group 1.

Results of multiple linear regression analysis with NAPCR as the dependent variable (Table 3) revealed that the NAPCR was associated with urinary biomarkers in total patients and in both groups, even after adjusting for confounding factors associated with diabetic kidney disease (age, gender, duration of diabetes, SBP, HbA1c, LDL and eGFR).

**Table 3:** Multivariate regression analysis with non-albumin protein-to-creatinine ratio as the dependent variable

	Total patients (424)			Group 1 (269)			Group 2 (155)		
	Model 1	Model 2	Model 3	Model 1	Model 2	Model 3	Model 1	Model 2	Model 3
	adj R^2^	adj R^2^	adj R^2^	adj R^2^	adj R^2^	adj R^2^	adj R^2^	adj R^2^	adj R^2^
ACR	0.089	0.103	0.419	0.234	0.511	0.566	0.157	0.160	0.412
*p* value	<0.001	<0.001	<0.001	<0.001	<0.001	<0.001	<0.001	<0.001	<0.001
PCR	0.055	0.066	0.379	0.211	0.332	0.401	0.091	0.105	0.415
*p* value	<0.001	<0.001	< 0.001	< 0.001	< 0.001	< 0.001	0.003	0.002	<0.001
Transferrin/Cr	0.012	0.016	0.400	0.041	0.079	0.126	0.035	0.028	0.335
*p* value	0.058	0.044	<0.001	0.004	<0.001	<0.001	0.070	0.127	<0.001
RBP/Cr	0.044	0.076	0.409	0.081	0.127	0.196	0.058	0.061	0.391
*p* value	<0.001	<0.001	< 0.001	< 0.001	< 0.001	< 0.001	0.023	0.028	<0.001
NGAL/Cr	0.053	0.051	0.361	0.019	0.020	0.059	0.134	0.135	0.427
*p* value	<0.001	<0.001	< 0.001	0.053	0.061	0.003	<0.001	<0.001	<0.001

Group 1, eGFR ≥60 mL/min/1.73 m^2^; Group 2, eGFR <60 mL/min/1.73 m^2^; *ACR* Albumin-to-creatinine ratio, *PCR* Total protein-to-creatinine ratio, Transferrin/Cr transferrin-to-creatinine ratio; *RBP/Cr* Retinol binding protein-to-creatinine ratio, *NGAL/Cr* Neutrophil gelatinase-associated lipocalin-to-creatinine ratio

Model 1, adjusted for age, gender and duration of diabetes

Model 2, adjusted for age, gender and duration of diabetes, SBP

Model 3, adjusted for age, gender, duration of diabetes, SBP, HbA1c, LDL and eGFR

Values of *p* < 0.05 were considered significant

The results of multiple linear regression analyses with PCR and ACR as the dependent variables are summarized in Additional file 3, Table S3 and Additional file 4, Table S4 respectively. Both the PCR and ACR were significantly associated with all urinary biomarkers in all patients and in all groups, even after adjusting for confounding factors.

**Fig. 2:**
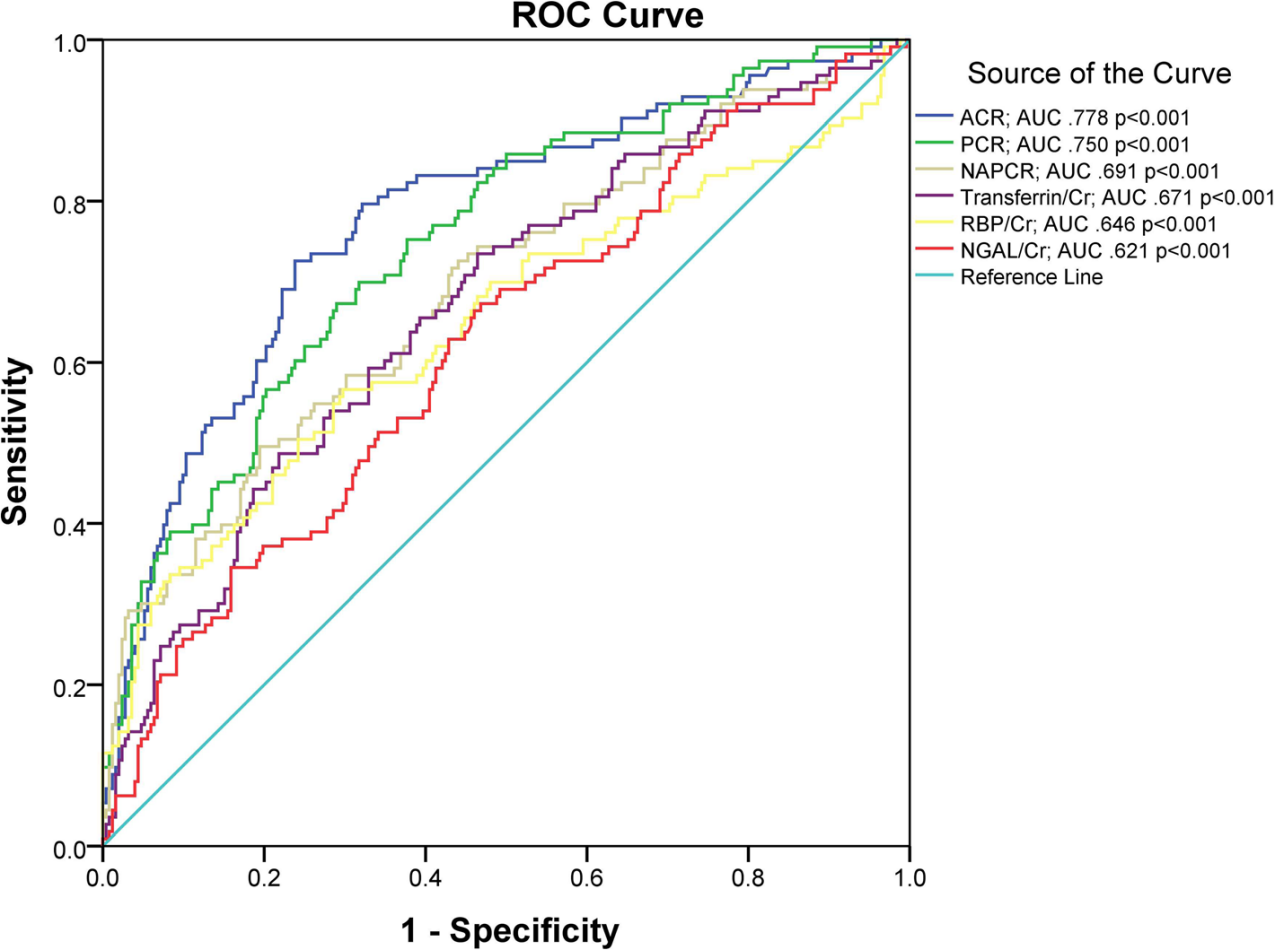
The receiver operating characteristics curves of different urinary biomarkers in type 2 diabetic patients with decreased eGFR levels

The diagnostic performance of various urinary biomarkers in patients with T2D with reduced eGFR (<60 mL/min/1.73 m^2^) are illustrated in Fig. 2. The AUC was largest for ACR. Although, the AUC for the NAPCR was larger than those of urinary markers for glomerular and tubular damage such as transferrin/Cr, RBP/Cr and NGAL/Cr. Additional file 5, Table S5 compares the ROC of different urinary markers in T2D. In the ROC comparison, both ACR and PCR show significant difference between NAPCR, Transferrin/Cr, RBP/Cr and NGAL/Cr. There were significant differences between NAPCR and tubular marker (NGAL/Cr) *p*=0.033 ROC curve areas in type 2 diabetes.

## Discussion

In this study, NAPCR was significantly correlated with PCR, glomerular (ACR, transferrin/Cr) and tubular (RBP/Cr and NGAL/Cr) markers. NAPCR in T2D with decreased eGFR diagnostically only outperformed NGAL/Cr.

The association of urinary NAP with tubular markers in normoalbuminuric patients has previously been reported, suggesting that urinary NAP can used as a marker for detecting tubular damage during the early stages of DN [21]. Previous studies reported that the urinary NAP level was a remarkable marker compared with albuminuria for predicting the progression of kidney diseases in diabetic patients, and the association of the NAPCR with eGFR showed the highest R^2^ value among all urinary markers that were evaluated [14, 15]. Although NAP is one component of urinary total protein, these studies did not analyze associations of the PCR with other urinary markers. In our study, the associations of PCR, ACR and NAPCR with different urinary markers were analyzed separately; which revealed that all three perform similarly after adjustment for different confounding factors, in both groups.

Several studies reported that urinary RBP and NGAL were associated with tubular damage that occurred during early stages of DN. The multivariate analysis revealed that NAPCR was associated with all the markers after adjusting for confounding factors. Even though, in Group 2, NAPCR was not significantly associated with transferrin/Cr after adjustment for confounding factors of age, gender, duration of diabetes, systolic blood pressure (SBP) in model 1 and 2. Urinary transferrin is considered a sensitive marker of glomerular damage, and a significant association with albumin excretion has been shown [22]. Urinary NAP comprises various tubular markers that are associated with renal impairment [23]. Taken together, these results suggest that NAP could be used as a marker for tubular damage in patients with T2D with severely decreased eGFR. Previous studies suggest that, among all the markers of tubular damage, NAPCR is the most appropriate for the evaluation of development and progression of renal diseases [24, 25]. Levels of RBP have been found to be associated with proximal tubular dysfunction that occurred independently of glomerular alteration and a weak association of RBP with urinary albumin excretion has been reported previously in patients with T2D [26, 27].

In this study ACR and PCR had better diagnostic value than other urinary biomarkers in T2D with decreased eGFR levels. These markers outperformed tubular markers such as RBP/Cr and NGAL/Cr in terms of diagnostic accuracy, in patients with T2D with decreased eGFR. Levels of urinary RBP were significantly higher in microalbuminuric diabetics when compared with normoalbuminuric and controls, indicating impaired proximal renal tubular function in early stage of DN [28, 29]. But in this study, no significant differences between NAPCR and RBP/Cr ROC curve areas. This might due to particularly urinary excretion of RBP4 was reported as highly specific for tubular disease in diabetics [30]. Unfortunately, in the current study we compared RBP/Cr with NAPCR instead of RBP4. A previous study supports current findings that NAPCR may be also a better predictor for the development and progression of chronic kidney disease in type 2 diabetic patients [14].

Previous study has demonstrated the potential of a multiple-biomarker approach for diagnosis of DN [31]. The use of such an approach can give additional insight into the condition of the patient, as DN is a multifactorial disease where several mechanisms are involved in disease activation. The RBP/Cr and NGAL/Cr reflect different pathophysiological pathways, and analysis of both can provide a better outcome in terms of the diagnosis and prognosis of DN. Despite the potential benefits, this may not be practical in routine clinical practice due to the increased cost and complexity of the procedure compared with single-biomarker approaches. However, estimation of urinary total protein and NAP is a more cost-effective approach than the immunological methodology required for the estimation of urinary transferrin, RBP and NGAL. Although NAP is calculated from urinary total protein levels, recent studies have not considered urinary total protein in their study and concluded NAPCR as a better predictor of renal impairment than other urinary biomarkers [14, 15]. As in the comparison of ROC curve of different urinary markers, NAPCR showed a significant difference only with tubular marker not with glomerular marker. Hence, it can be consider as a confirmative cost-effective biomarker along with PCR in the diagnosis of tubular injury in T2D with decreased eGFR.The limitations of this study include, the study was not based on the longitudinal observations but was conducted with a cross sectional design. Further investigations are necessary in order to determine the diagnostic utility of different biomarkers. The examinations were confined to Saudi patients with T2D and therefore the results may not be able to be generalized to other ethnicities. Second, the disease condition of study participants was unclear in terms of the presence of glomerular and tubular damage, or a combination of both. Finally, the effect of drugs on either proteinuria or eGFR was not considered because study patients had histories of > 10 years of DM and presented with diabetic complications, including vasculopathy, retinopathy, hypertension, and hyperlipidemia. These conditions are prevalent among diabetic patients having longer duration and the majority of our study patients were prescribed drugs to manage their complications. The changes in biomarker levels due to the effects of these drugs will therefore be similar for study participants, and we can conclude that this will not have affected our results.

## Conclusions

This study demonstrates that ACR and PCR are diagnostic biomarkers in T2D patients with decreased eGFR. Nevertheless, these results also suggest that along with ACR and PCR, NAP may provide additional value for diagnosis of tubular damage in diabetic patients with decreased eGFR levels. Furthermore, this preliminary study will clearly need a validation with future prospective analysis on larger sample size.

## Supplementary information

**Additional file 1: Table S1.** Correlation of total protein-to-creatinine ratio with clinical, anthropometric and biochemical characteristics and other urinary markers in type 2 diabetes subjects. Note. Group 1,eGFR ≥60 mL/min/1.73 m^2^; Group 2, eGFR < 60 mL/min/1.73 m^2^; BMI, body mass index; SBP, systolic blood pressure; DBP, diastolic blood pressure; FBS, fasting blood sugar; HbA1c, hemoglobin A1c; LDL, low-density lipoprotein; HDL, high-density lipoprotein; eGFR, estimated glomerular filtration rate; ACR, albumin-to-creatinine ratio; NAPCR, non-albumin protein-to-creatinine ratio; Transferrin/Cr, transferrin-to-creatinine ratio; RBP/Cr, retinol binding protein-to-creatinine ratio; NGAL/Cr, neutrophil gelatinase-associated lipocalin-to-creatinine ratio; r, coefficients of correlation; values of *p* < 0.05 were considered significant.

**Additional file 2: Table S2.** Correlation of albumin-to-creatinine ratio with clinical, anthropometric and biochemical characteristics and other urinary markers in type 2 diabetes subjects. Note. Group 1,eGFR ≥60 mL/min/1.73 m^2^; Group 2, eGFR < 60 mL/min/1.73 m^2^; BMI, body mass index; SBP, systolic blood pressure; DBP, diastolic blood pressure; FBS, fasting blood sugar; HbA1c, hemoglobin A1c; LDL, low-density lipoprotein; HDL, high-density lipoprotein; eGFR, estimated glomerular filtration rate; PCR, total protein-to-creatinine ratio; NAPCR, non-albumin protein-to-creatinine ratio; Transferrin/Cr, transferrin-to-creatinine ratio; RBP/Cr, retinol binding protein-to-creatinine ratio; NGAL/Cr, neutrophil gelatinase-associated lipocalin-to-creatinine ratio; r, coefficients of correlation; values of *p* < 0.05 were considered significant.

**Additional file 3: Table S3.** Multivariate regression analysis with total protein-to-creatinine ratio as the dependent variable. Note. Group 1, eGFR ≥60 mL/min/1.73 m^2^; Group 2, eGFR < 60 mL/min/1.73 m^2^; ACR, albumin-to-creatinine ratio; NAPCR, non-albumin protein-to-creatinine ratio; Transferrin/Cr, transferrin-to-creatinine ratio; RBP/Cr, retinol binding protein-to-creatinine ratio; NGAL/Cr, neutrophil gelatinase-associated lipocalin-to-creatinine ratio. Model 1, adjusted for age, gender and duration of diabetes. Model 2, adjusted for age, gender and duration of diabetes, SBP. Model 3, adjusted for age, gender, duration of diabetes, SBP, HbA1c, LDL and eGFR. Values of *p* < 0.05 were considered significant.

**Additional file 4: Table S4.** Multivariate regression analysis with albumin-to-creatinine ratio as the dependent variable. Note. Group 1, eGFR ≥60 mL/min/1.73 m^2^; Group 2, eGFR < 60 mL/min/1.73 m^2^; PCR, total protein-to-creatinine ratio; NAPCR, non-albumin protein-to-creatinine ratio; Transferrin/Cr, transferrin-to-creatinine ratio; RBP/Cr, retinol binding protein-to-creatinine ratio; NGAL/Cr, neutrophil gelatinase-associated lipocalin-to-creatinine ratio. Model 1, adjusted for age, gender and duration of diabetes. Model 2, adjusted for age, gender and duration of diabetes, SBP. Model 3, adjusted for age, gender, duration of diabetes, SBP, HbA1c, LDL and eGFR. Values of *p* < 0.05 were considered significant.

**Additional file 5: Table S5.** The comparison of ROC curves of different urinary markers in type 2 diabetes subjects. Note. ACR, albumin-to-creatinine ratio; PCR, total protein-to-creatinine ratio; NAPCR, non-albumin protein-to-creatinine ratio; Transferrin/Cr, transferrin-to-creatinine ratio; RBP/Cr, retinol binding protein-to-creatinine ratio; NGAL/Cr, neutrophil gelatinase-associated lipocalin-to-creatinine ratio; values of *p* < 0.05 were considered significant.

## Data Availability

The data that support the findings of this study are available from King Abdulaziz City for Science and Technology (KACST), but restrictions apply to the availability of these data, which were used under license for the current study, and so are not publicly available. Data are however available from the authors upon reasonable request and with permission of King Abdulaziz City for Science and Technology (KACST).

## Abbreviations

NAP: Non albumin protein
T2D: Type 2 diabetes
eGFR: Estimated glomerular filtration rate
RBP: Retinol binding protein
NGAL: Neutrophil gelatinase-associated lipocalin
ACR: Albumin-to-creatinine ratio
NAPCR: Non-albumin protein-to-creatinine ratio
PCR: Total protein-to-creatinine ratio
DM: Diabetes mellitus
DN: Diabetic nephropathy
IRB: Institutional Review Board
STARD: Standards for reporting diagnostic accuracy studies
FBS: Fasting blood sugar
HDL: High-density lipoprotein
LDL: Low-density lipoprotein
TC: Total cholesterol
BMI: Body mass index
SBP: Systolic blood pressure
DBP: Diastolic blood pressure
ELISA: Enzyme-linked immunosorbent assay
IQR: Interquartile range
AUC: Area under curve
ROC: Receiver operating characteristics
RBP/Cr: Retinol binding protein-to-creatinine ratio
NGAL/Cr: Neutrophil gelatinase-associated lipocalin-to-creatinine ratio
