# Supplementary material for: Association of urinary non-albumin protein with the different urinary marker for glomerular and tubular damage in patients with type 2 diabetes
Source: BMC Nephrol. 2020 Jul 6;21:255. doi: 10.1186/s12882-020-01906-6 (PMC7336477; doi:10.1186/s12882-020-01906-6)
Supplement: Supplementary file 1 — Additional file 1: Table S1. Correlation of total protein-to-creatinine ratio with clinical, anthropometric and biochemical characteristics and other urinary markers in type 2 diabetes subjects. Note. Group 1,eGFR ≥60 mL/min/1.73 m2; Group 2, eGFR < 60 mL/min/1.73 m2; BMI, body mass index; SBP, systolic blood pressure; DBP, diastolic blood pressure; FBS, fasting blood sugar; HbA1c, hemoglobin A1c; LDL, low-density lipoprotein; HDL, high-density lipoprotein; eGFR, estimated glomerular filtration rate; ACR, albumin-to-creatinine ratio; NAPCR, non-albumin protein-to-creatinine ratio; Transferrin/Cr, transferrin-to-creatinine ratio; RBP/Cr, retinol binding protein-to-creatinine ratio; NGAL/Cr, neutrophil gelatinase-associated lipocalin-to-creatinine ratio; r, coefficients of correlation; values of p < 0.05 were considered significant. [file 12882_2020_1906_MOESM1_ESM.pdf]

Additional file 1: Table S1. Correlation of total protein-to-creatinine ratio with clinical, anthropometric and biochemical characteristics and other urinary markers in type 2 diabetes subjects.

Variables	Total patients (424)		Group 1 (269)		Group 2 (155)	
	r	*p*	r	*p*	r	*p*
Age years	0.063	0.221	0.072	0.254	-0.014	0.877
BMI, kg/m^2	0.016	0.756	0.030	0.630	-0.013	0.887
Duration of diabetes, years	0.113	0.029	0.154	0.014	0.066	0.476
SBP, mmHg	0.161	0.002	0.234	<0.001	0.092	0.320
DBP, mmHg	-0.001	0.990	0.137	0.029	-0.097	0.298
FBS, mg/dL	0.083	0.111	0.136	0.030	0.001	0.999
HbA1c, %	0.242	<0.001	0.234	<0.001	0.231	0.012
Total cholesterol, mg/dL	0.073	0.160	0.166	0.008	-0.089	0.339
LDL, mg/dL	-0.010	0.853	0.105	0.094	-0.116	0.215
HDL, mg/dL	0.016	0.765	0.090	0.153	-0.066	0.480
Triglycerides, mg/dL	0.076	0.146	0.171	0.006	-0.067	0.479
eGFR, mL/min/1.73 m^2	-0.465	<0.001	-0.238	<0.001	-0.570	<0.001
ACR, mg/g	0.895	<0.001	0.808	<0.001	0.898	<0.001
NAPCR, mg/g	0.873	<0.001	0.850	<0.001	0.867	<0.001
Transferrin/Cr, µg/g	0.583	<0.001	0.458	<0.001	0.587	<0.001
RBP/Cr, µg/g	0.725	<0.001	0.635	<0.001	0.721	<0.001
NGAL/Cr, µg/g	0.441	<0.001	0.192	0.002	0.579	<0.001

Group 1, eGFR ≥60 mL/min/1.73 m^2; Group 2, eGFR <60 mL/min/1.73 m^2; BMI, body mass index; SBP, systolic blood pressure; DBP, diastolic blood pressure; FBS, fasting blood sugar; HbA1c, hemoglobin A1c; LDL, low-density lipoprotein; HDL, high-density lipoprotein; eGFR, estimated glomerular filtration rate; ACR, albumin-to-creatinine ratio; NAPCR, non-albumin protein-to-creatinine ratio; Transferrin/Cr, transferrin-to-creatinine ratio; RBP/Cr, retinol binding protein-to-creatinine ratio; NGAL/Cr, neutrophil gelatinase-associated lipocalin-to-creatinine ratio; r, coefficients of correlation; values of $p < 0.05$ were considered significant.
