# Supplementary material for: Association of urinary non-albumin protein with the different urinary marker for glomerular and tubular damage in patients with type 2 diabetes
Source: BMC Nephrol. 2020 Jul 6;21:255. doi: 10.1186/s12882-020-01906-6 (PMC7336477; doi:10.1186/s12882-020-01906-6)
Supplement: Supplementary file 2 — Additional file 2: Table S2. Correlation of albumin-to-creatinine ratio with clinical, anthropometric and biochemical characteristics and other urinary markers in type 2 diabetes subjects. Note. Group 1,eGFR ≥60 mL/min/1.73 m2; Group 2, eGFR < 60 mL/min/1.73 m2; BMI, body mass index; SBP, systolic blood pressure; DBP, diastolic blood pressure; FBS, fasting blood sugar; HbA1c, hemoglobin A1c; LDL, low-density lipoprotein; HDL, high-density lipoprotein; eGFR, estimated glomerular filtration rate; PCR, total protein-to-creatinine ratio; NAPCR, non-albumin protein-to-creatinine ratio; Transferrin/Cr, transferrin-to-creatinine ratio; RBP/Cr, retinol binding protein-to-creatinine ratio; NGAL/Cr, neutrophil gelatinase-associated lipocalin-to-creatinine ratio; r, coefficients of correlation; values of p < 0.05 were considered significant. [file 12882_2020_1906_MOESM2_ESM.pdf]

Additional file 2: Table S2. Correlation of albumin-to-creatinine ratio with clinical, anthropometric and biochemical characteristics and other urinary markers in type 2 diabetes subjects.

**Variables**	**Total patients (424)**		**Group 1 (269)**		**Group 2 (155)**	
	r	*p*	r	*p*	r	*p*
Age years	0.001	0.979	0.060	0.327	-0.087	0.280
BMI, kg/m^2	-0.011	0.816	-0.028	0.648	-0.022	0.790
Duration of diabetes, years	0.146	0.003	0.132	0.031	0.138	0.087
SBP, mmHg	0.203	<0.001	0.206	0.001	0.176	0.028
DBP, mmHg	-0.041	0.402	0.051	0.403	-0.091	0.258
FBS, mg/dL	0.089	0.068	0.158	0.010	0.014	0.865
HbA1c, %	0.195	<0.001	0.186	0.002	0.186	0.020
Total cholesterol, mg/dL	0.065	0.185	0.172	0.005	-0.079	0.328
LDL, mg/dL	0.009	0.860	0.113	0.066	-0.059	0.472
HDL, mg/dL	0.006	0.907	0.028	0.653	-0.059	0.472
Triglycerides, mg/dL	0.174	<0.001	0.251	<0.001	0.088	0.284
eGFR, mL/min/1.73 m^2	-0.457	<0.001	-0.222	<0.001	-0.482	<0.001
PCR, mg/g	0.895	<0.001	0.808	<0.001	0.898	<0.001
NAPCR, mg/g	0.624	<0.001	0.411	<0.001	0.635	<0.001
Transferrin/Cr, µg/g	0.473	<0.001	0.465	<0.001	0.446	<0.001
RBP/Cr, µg/g	0.578	<0.001	0.400	<0.001	0.541	<0.001
NGAL/Cr, µg/g	0.323	<0.001	0.054	0.382	0.323	<0.001

Group 1,eGFR ≥60 mL/min/1.73 m^2; Group 2, eGFR <60 mL/min/1.73 m^2; BMI, body mass index; SBP, systolic blood pressure; DBP, diastolic blood pressure; FBS, fasting blood sugar; HbA1c, hemoglobin A1c; LDL, low-density lipoprotein; HDL, high-density lipoprotein; eGFR, estimated glomerular filtration rate; PCR, total protein-to-creatinine ratio; NAPCR, non-albumin protein-to-creatinine ratio; Transferrin/Cr, transferrin-to-creatinine ratio; RBP/Cr, retinol binding protein-to-creatinine ratio; NGAL/Cr, neutrophil gelatinase-associated lipocalin-to-creatinine ratio; r, coefficients of correlation; values of $p < 0.05$ were considered significant.
