# Supplementary material for: Association of urinary non-albumin protein with the different urinary marker for glomerular and tubular damage in patients with type 2 diabetes
Source: BMC Nephrol. 2020 Jul 6;21:255. doi: 10.1186/s12882-020-01906-6 (PMC7336477; doi:10.1186/s12882-020-01906-6)
Supplement: Supplementary file 4 — Additional file 4: Table S4. Multivariate regression analysis with albumin-to-creatinine ratio as the dependent variable. Note. Group 1, eGFR ≥60 mL/min/1.73 m2; Group 2, eGFR < 60 mL/min/1.73 m2; PCR, total protein-to-creatinine ratio; NAPCR, non-albumin protein-to-creatinine ratio; Transferrin/Cr, transferrin-to-creatinine ratio; RBP/Cr, retinol binding protein-to-creatinine ratio; NGAL/Cr, neutrophil gelatinase-associated lipocalin-to-creatinine ratio. Model 1, adjusted for age, gender and duration of diabetes. Model 2, adjusted for age, gender and duration of diabetes, SBP. Model 3, adjusted for age, gender, duration of diabetes, SBP, HbA1c, LDL and eGFR. Values of p < 0.05 were considered significant. [file 12882_2020_1906_MOESM4_ESM.pdf]

Additional file 4: Table S4. Multivariate regression analysis with albumin-to-creatinine ratio as the dependent variable.

	**Total patients (424)**			**Group 1 (269)**			**Group 2 (155)**		
	Model 1 adj R^2	Model 2 adj R^2	Model 3 adj R^2	Model 1 adj R^2	Model 2 adj R^2	Model 3 adj R^2	Model 1 adj R^2	Model 2 adj R^2	Model 3 adj R^2
PCR	0.130	0.158	0.398	0.001	0.134	0.288	0.169	0.185	0.309
p value	<0.001	<0.001	<0.001	0.407	<0.001	<0.001	<0.001	<0.001	<0.001
NAPCR	0.139	0.172	0.470	0.096	0.457	0.520	0.222	0.229	0.436
p value	<0.001	<0.001	<0.001	<0.001	<0.001	<0.001	<0.001	<0.001	<0.001
Transferrin/Cr	0.024	0.117	0.380	0.003	0.151	0.253	0.028	0.093	0.260
p value	0.005	<0.001	<0.001	0.277	<0.001	<0.001	0.067	0.001	<0.001
RBP/Cr	0.034	0.122	0.378	0.042	0.071	0.145	0.030	0.063	0.181
p value	0.001	<0.001	<0.001	0.003	<0.001	<0.001	0.063	0.010	<0.001
NGAL/Cr	0.044	0.102	0.464	0.017	0.032	0.055	0.058	0.069	0.415
p value	<0.001	<0.001	<0.001	0.055	0.014	0.003	0.008	0.006	<0.001

Group 1, eGFR ≥60 mL/min/1.73 m^2; Group 2, eGFR <60 mL/min/1.73 m^2; PCR, total protein-to-creatinine ratio; NAPCR, non-albumin protein-to-creatinine ratio; Transferrin/Cr, transferrin-to-creatinine ratio; RBP/Cr, retinol binding protein-to-creatinine ratio; NGAL/Cr, neutrophil gelatinase-associated lipocalin-to-creatinine ratio.

Model 1, adjusted for age, gender and duration of diabetes.
Model 2, adjusted for age, gender and duration of diabetes, SBP.
Model 3, adjusted for age, gender, duration of diabetes, SBP, HbA1c, LDL and eGFR.
Values of $p < 0.05$ were considered significant.
