# Supplementary material for: Association of urinary non-albumin protein with the different urinary marker for glomerular and tubular damage in patients with type 2 diabetes
Source: BMC Nephrol. 2020 Jul 6;21:255. doi: 10.1186/s12882-020-01906-6 (PMC7336477; doi:10.1186/s12882-020-01906-6)
Supplement: Supplementary file 5 — Additional file 5: Table S5. The comparison of ROC curves of different urinary markers in type 2 diabetes subjects. Note. ACR, albumin-to-creatinine ratio; PCR, total protein-to-creatinine ratio; NAPCR, non-albumin protein-to-creatinine ratio; Transferrin/Cr, transferrin-to-creatinine ratio; RBP/Cr, retinol binding protein-to-creatinine ratio; NGAL/Cr, neutrophil gelatinase-associated lipocalin-to-creatinine ratio; values of p < 0.05 were considered significant. [file 12882_2020_1906_MOESM5_ESM.pdf]

Additional file 5: Table S5. The comparison of ROC curves of different urinary markers in type 2 diabetes subjects

Pairwise comparison of ROC curves	Difference between areas	Standard Error [a]	95% Confidence Interval	z statistic	*p*
ACR~PCR	0.028	0.021	-0.013 – 0.070	1.334	0.182
ACR~NAPCR	0.086	0.032	0.024 – 0.149	2.709	0.006
ACR~Transferrin/Cr	0.107	0.023	0.060 – 0.154	4.500	<0.0001
ACR~RBP/Cr	0.132	0.034	0.064 – 0.200	3.805	0.0001
ACR~NGAL/Cr	0.157	0.036	0.085 – 0.228	4.289	<0.0001
PCR~NAPCR	0.058	0.015	0.028 – 0.087	3.852	0.0001
PCR~Transferrin/Cr	0.078	0.023	0.032 – 0.126	3.309	0.0009
PCR~RBP/Cr	0.104	0.028	0.048 – 0.159	3.665	0.0002
PCR~NGAL/Cr	0.128	0.032	0.067 – 0.190	4.111	<0.0001
NAPCR~Transferrin/Cr	0.020	0.029	-0.037 – 0.078	0.698	0.485
NAPCR~RBP/Cr	0.045	0.030	-0.013 – 0.105	1.503	0.132
NAPCR~NGAL/Cr	0.070	0.033	0.005 – 0.135	2.127	0.033
Transferrin/Cr~RBP/Cr	0.024	0.031	-0.036 –0.085	0.802	0.422
Transferrin/Cr~NGAL/Cr	0.049	0.034	-0.017 – 0.116	1.450	0.147
RBP/Cr~NGAL/Cr	0.024	0.033	-0.041– 0.090	0.726	0.467

ACR, albumin-to-creatinine ratio; NAPCR, non-albumin protein-to-creatinine ratio; Transferrin/Cr, transferrin-to-creatinine ratio; RBP/Cr, retinol binding protein-to-creatinine ratio; NGAL/Cr, neutrophil gelatinase-associated lipocalin-to-creatinine ratio; values of $p < 0.05$ were considered significant.
